# Brain‐to‐Brain Coupling Is Associated With the Benefits of Delayed Imitation in Skill Acquisition: An fNIRS Study

**DOI:** 10.1002/brb3.71683

**Published:** 2026-08-20

**Authors:** Cuirong Yang, Linwei Yu, Liangchuan Mi, Ming Yin, Yi Hu

**Affiliations:** ^1^ School of Smart Education Jiangsu Normal University Xuzhou China; ^2^ Shanghai Key Laboratory of Mental Health and Psychological Crisis Intervention School of Psychology and Cognitive Science East China Normal University Shanghai China; ^3^ School of Water Conservancy Engineering Zhejiang Tongji Vocational College of Science and Technology Hangzhou China; ^4^ Investigation Department Jiangsu Police Institute Nanjing China

**Keywords:** delayed imitation, fNIRS, interbrain synchrony, observation learning

## Abstract

**Purpose:**

Observational learning through imitation is an important ability across various domains. Although research has found that delayed imitation produces superior learning outcomes compared with other observational learning strategies, the underlying reason for this advantage remains unclear. This study investigated whether interpersonal brain synchronization (IBS) between learners and demonstrators is related to the benefits of delayed imitation.

**Methods:**

A total of 48 pairs of participants (M_age_ = 19.44, SD_age_ = 0.71) engaged in an abacus learning task under three learning conditions: observation without imitation, concurrent imitation, and delayed imitation. The brain activity of learners and demonstrators was simultaneously recorded using functional near‐infrared spectroscopy.

**Results:**

Delayed imitation led to better learning outcomes than concurrent imitation across both skill operation performance and operational rules. However, performance in the delayed imitation condition did not differ significantly from that in the observation‐only condition. At the neural level, delayed imitation elicited stronger IBS in the right dlPFC, with both observation and imitation phases contributing to this increased synchronization. Crucially, IBS in the right dlPFC was positively correlated with learning performance.

**Conclusions:**

These findings suggest that the advantage of delayed imitation is associated with enhanced brain‐to‐brain coupling between the demonstrator and the learner, providing novel evidence that interbrain coupling contributes to effective observational learning.

## Graphical abstract

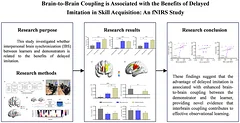

## 1 Introduction

Learning by observing and imitating others is a fundamental ability. This process, known as observational learning, allows individuals to acquire new behaviors or skills (Bandura 1977). Many studies in educational psychology have demonstrated the benefits of observational learning, which helps people master motor skills (Francisco et al. 2023), gain knowledge (Zhidan, Williamson, et al. 2015), understand rules (Zhidan, Meltzoff, et al. 2015), and form social attitudes (Skinner et al. 2020). Observational learning typically involves both observation and imitation. This means that observational learning is not limited to passively watching others; rather, it often requires learners to imitate the observed actions. Accordingly, observational learning is sometimes referred to as imitation learning (Iacoboni et al. 1999; Meltzoff and Williamson 2013). Observational learning can take different forms, including delayed imitation learning, concurrent imitation learning, and observation‐only learning without overt imitation (Krüger et al. 2014; Weeks et al. 1996). Delayed imitation learning adopts a strategy in which learners imitate after observing, while concurrent imitation learning entails learners imitating demonstration actions while observing them (Crone et al. 2021).

Previous studies have demonstrated that different imitation strategies produce varied learning outcomes. Specifically, many studies have found that delayed imitation yields better learning outcomes than concurrent imitation (Crone et al. 2021; Weeks et al. 1996). The prevailing explanation for this advantage centers on the allocation of cognitive resources. Concurrent imitation learning imposes a higher cognitive load by requiring learners to process visual information while coordinating their imitative actions. This demand can interfere with the initial encoding process, resulting in a less detailed or accurate memory of the demonstrated behavior. In contrast, delayed imitation learning allows learners to focus their attention on observation and retain the demonstrated behavior without the competing demands of concurrent execution during the observation stage (Crone et al. 2021; Weeks et al. 1996).

While this perspective explains the difference between delayed and concurrent imitation, it may not fully account for the relationship between delayed imitation and observation‐only learning. Observation‐only learning, like delayed imitative learning, does not require learners to replicate the demonstrated actions during the observation stage. Some studies have found comparable performance between delayed imitation learning and observation‐only learning, suggesting that uninterrupted observation phases may be the critical factor for successful learning (Fiorella et al. 2017; Sweller et al. 2020). However, many other studies have demonstrated that delayed imitation learning results in significantly better performance than observation‐only learning (Blandin et al. 1999; Cherdieu et al. 2017; Deakin and Proteau 2000; Zhidan, Williamson, et al. 2015). This may be because overt imitation triggers the activation of stored representations of the demonstrated behaviors in memory due to shared sensorymotor representations (Iacoboni 2009; Iacoboni et al. 1999), leading to better performance. These findings suggest that the benefit of delayed imitation learning cannot be attributed solely to observation. In fact, in the context of delayed imitation, the observation and imitation phases form a continuous cognitive loop rather than isolated events (Bandura 1977). During the observation phase of delayed imitation, learners actively encode the demonstrator's actions and maintain these mental representations for later execution. During the subsequent imitation phase, learners execute the actions while continuously retrieving and comparing their performance with the stored memory of the demonstration (Bandura 1977; Meltzoff 2005, 2007). Because these cognitive processes (encoding, retention, and execution) are highly interdependent, the neural coupling between the demonstrator and learner is sustained across the entire learning episode. Thus, this integrated process likely manifests as stronger interpersonal brain synchronization (IBS) throughout the entire learning episode, rather than emerging solely within specific sub‐phases.

Current research primarily focuses on individual cognitive processes to explain the impact of delayed imitation on learning outcomes, largely overlooking the social process of observational learning. Given that observational learning typically occurs within teaching contexts, the interaction between learners and demonstrators may play an important role in the process. Recent studies in social neuroscience suggest that brain coupling may facilitate communication and understanding during the learning process (Hasson et al. 2012; Hasson et al. 2004; Pan et al. 2020). However, it remains unclear how different imitation strategies modulate brain‐to‐brain coupling and whether this neural coupling can account for the benefits of delayed imitation. This study, therefore, aims to investigate the IBS between learners and demonstrators and examine its correlation with learning outcomes under different observational learning strategies. By examining brain‐to‐brain coupling, this research seeks to offer novel insights into the mechanisms of effective observational learning.

Previous research provides a foundation for understanding how brain‐to‐brain coupling is related to learning performance. Studies examining teacher‐student dyads have demonstrated that more effective instructional approaches are associated with enhanced IBS in the prefrontal cortex between teachers and students. For instance, face‐to‐face communication between teachers and students with relevant prior knowledge results in greater IBS in the left prefrontal cortex and better learning performance than computer‐based communication (Liu et al. 2019). Similarly, in the context of scaffolding strategies, enhanced IBS in the prefrontal cortex is positively associated with learning outcomes (Pan et al. 2020). Furthermore, elaborative feedback between teachers and students was linked to enhanced IBS in the parietal cortex and improved learning outcomes (Zhu et al. 2022). These findings suggest that brain synchronization in the prefrontal and parietal cortices may be positively correlated with better learning outcomes.

The prefrontal cortex plays a crucial role in cognitive control and execution (Friedman and Robbins 2022; Miller and Cohen 2001), and also supports social interaction and the learning process (Hasson et al. 2012; Hasson et al. 2004) during imitative learning. Importantly, delayed imitation places distinct cognitive demands on these neural systems compared to other observational learning strategies. During delayed imitation learning, learners must encode and maintain action representations in working memory, plan upcoming movements, and monitor the accuracy of subsequent execution. Single‐brain neuroimaging studies have found that delayed imitation learning increases activation of the prefrontal cortex, especially the dlPFC, due to additional cognitive processes, including planning, encoding, intentional processing, comprehending demonstrated actions, and monitoring imitated actions (Higuchi et al. 2012; Vogt et al. 2007). The parietal cortex also exhibits increased activation during the observational phase of delayed imitation learning (Krüger et al. 2014; Vogt et al. 2007) and is reciprocally interconnected with the prefrontal cortex to support imitative learning (Iacoboni et al. 1999). This sustained neural activity suggests that learners maintain detailed action representations while preparing for subsequent reproduction, potentially enhancing neural coupling between the demonstrator and the learner. In contrast, the dual‐task demands of concurrent imitation may limit the depth of neural engagement, potentially reducing the strength of interpersonal synchronization. Taken together, these findings suggest that delayed imitation learning may evoke enhanced IBS in the prefrontal and parietal cortices compared with other observational learning strategies throughout the learning process.

In addition, the first‐person viewpoint of a demonstration has been shown to elicit stronger activation in the MNS than the third‐person viewpoint (Jackson et al. 2006; Leng et al. 2024; Watanabe et al. 2017). Accordingly, observing modeling videos from a first‐person perspective is more effective for learning than observing them from a third‐person perspective (de Koning et al. 2023; Fiorella et al. 2017; Garland and Sanchez 2013) because processing third‐person viewpoints requires mental transformation into a first‐person viewpoint, adding to cognitive load (Kessler and Thomson 2010; Samuel et al. 2024). However, this perspective effect has not been consistently demonstrated for imitation accuracy (Boucheix et al. 2018; Leng et al. 2024) as imitation accuracy is also influenced by other variables such as the complexity of the demonstrated movements (Boucheix et al. 2018) and imitation intention (Barhorst‐Cates et al. 2022). The present study, therefore, also examines whether the viewpoint modulates the effect of imitation strategies on learning outcomes and IBS between learners and demonstrators.

### 1.1 Present Study

In summary, this study examines the mechanisms underlying the effects of different observational learning strategies on skill learning outcomes. We used an abacus calculation task as the experimental material and recorded the brain activity of both the demonstrator and the learner simultaneously using functional near‐infrared spectroscopy (fNIRS). This approach allowed us to examine IBS as participants engaged in delayed imitation learning, concurrent imitation learning, and mere observation‐only learning. Based on observational learning theory and related research findings (Bandura 1977; Meltzoff 2005, 2007), we hypothesized that delayed imitation learning would elicit stronger IBS than the other two strategies, owing to the interdependent nature of its observation and imitation stages. We further predicted that these higher levels of IBS would be associated with better learning outcomes. Ultimately, this study seeks to provide novel insights into how social brain dynamics contribute to effective observational learning.

## 2 Method

### 2.1 Participants

A total of 96 healthy participants with no prior abacus experience were recruited for the study and paired to form 48 same‐gender dyads. The dyads were randomly assigned to one of three groups, with 16 dyads per group: (1) observation‐only group (16 dyads including 2 female dyads, M_age_ = 19.94, SD_age_ = 1.12); (2) concurrent imitation group (16 dyads including 1 female dyad, M_age_ = 19.63, SD_age_ = 1.02); and (3) delayed imitation group (16 dyads including 1 female dyad, M_age_ = 19.56, SD_age_ = 1.03).

To evaluate the adequacy of the sample size, we conducted a sensitivity analysis using G*Power 3.1. For a 3 × 2 mixed‐design ANOVA (*α* = 0.05, 1−*β* = 0.80), the analysis indicated that the minimum detectable effect size was Cohen's *f* = 0.40 for the between‐subjects main effect, *f* = 0.21 for the within‐subjects main effect, and *f* = 0.23 for the interaction effect. The actual effect sizes observed for our main findings generally exceeded these theoretical thresholds. For instance, the significant main effects of imitation type (between‐subjects) on behavioral outcomes and IBS yielded partial *η* ^2^ values ranging from 0.31 to 0.60, corresponding to Cohen's *f* values ranging from 0.67 to 1.22. These comparisons suggest that our sample size of 48 dyads provided adequate statistical power to detect the primary effects of interest in the current study. Furthermore, this sample size aligns with previous fNIRS hyperscanning studies that reported significant interpersonal synchrony effects using comparable or smaller samples (Fronda and Balconi 2020; Holper et al. 2012; Shadaydeh et al. 2025).

In each group, one participant was randomly assigned as the instructor and the other as the learner. Because all participants were recruited without any abacus experience, the instructors received training before the formal experiment to ensure adequate abacus knowledge and skills. All participants provided informed consent before the experiment and received compensation of either 20 RMB or 40 RMB, depending on their role, after completing the experiment. No data from any dyad were excluded, as all participants completed the full experimental procedure.

### 2.2 Experimental Factors

This study used a two‐factor mixed design with observational learning type as the between‐subjects factor (observation‐only vs. concurrent imitation vs. delayed imitation) and with learner perspective as the within‐subjects factor (the first‐person viewpoint vs. the third‐person viewpoint). Learners in the observation‐only condition watched the instructor's modeling without imitation throughout the learning process. Learners in the concurrent imitation condition observed and imitated the instructor's modeling actions at the same time. Learners in the delayed imitation condition watched the instructor's actions first and then imitated them. In the first‐person viewpoint condition, the learner sat side by side with the instructor, sharing the same perspective. In the third‐person viewpoint condition, the learner sat on the opposite side of the instructor, obtaining a mirrored view of the demonstration.

### 2.3 Task and Learning Materials

Abacus skill was chosen as the task for observational learning because it enables individuals to perform complex arithmetic operations efficiently, despite being an old‐fashioned calculation method. Even today, undergraduates majoring in accounting, finance, and economics at Chinese universities are expected to acquire abacus skills. Participants who played the role of learner in this study were asked to learn basic abacus addition and subtraction skills by observing either nine addition or nine subtraction operations on the abacus (Sun 2022). Each abacus skill has a distinct abacus rhyme, making it possible to learn each skill independently and avoid any potential influence of knowledge relatedness on learning outcomes (See Table 1).

**TABLE 1:** Learning materials for the abacus observational learning task.

	Learning task	Mathematics formula	Abacus rhyme
Abacus addition	Direct addition	1 + 2	Add two directly if the addend is two. “二上二”
		2 + 2	Add two directly if the addend is two. “二上二”
		5 + 3	Add three directly if the addend is three. “三上三”
	Five complement addition	4 + 2	Subtract three, and add five if the addend is two. “二下五去三”
		4 + 3	Subtract two, and add five if the addend is three. “三下五去二”
		4 + 4	Subtract one, and add five if the addend is four. “四下五去一”
	Ten‐plus addition	9 + 2	Subtract eight, and carry over to the tenth place if the addend is two. “二去八进一”
		9 + 3	Subtract seven, and carry over to the tenth place if the addend is three. “三去七进一”
		9 + 4	Subtract six, and carry over to the tenth place if the addend is four. “四去六进一”
Abacus subtraction	Direct subtraction	2 − 1	Subtract one directly if the subtrahend is one. “一下一”
		2 − 2	Subtract two directly if the subtrahend is two. “二下二”
		8 − 3	Subtract three directly if the subtrahend is three. “三下三”
	Five break subtraction	5 − 2	Add three, and remove five, if the subtrahend is two. “二上三去五”
		5 − 3	Add two, and remove five, if the subtrahend is three. “三上二去五”
		6 − 4	Add one, and remove five, if the subtrahend is four. “四上一去五”
	Ten‐borrowing subtraction	11 − 4	Add six, and borrow from the tenth place if the subtrahend is four. “四退一还六”
		11 − 5	Add five, and borrow from the tenth place if the subtrahend is five “五退一还五”
		11 − 6	Add four, and borrow from the tenth place if the subtrahend is six. “六退一还四”

### 2.4 Procedure

**FIGURE 1:**
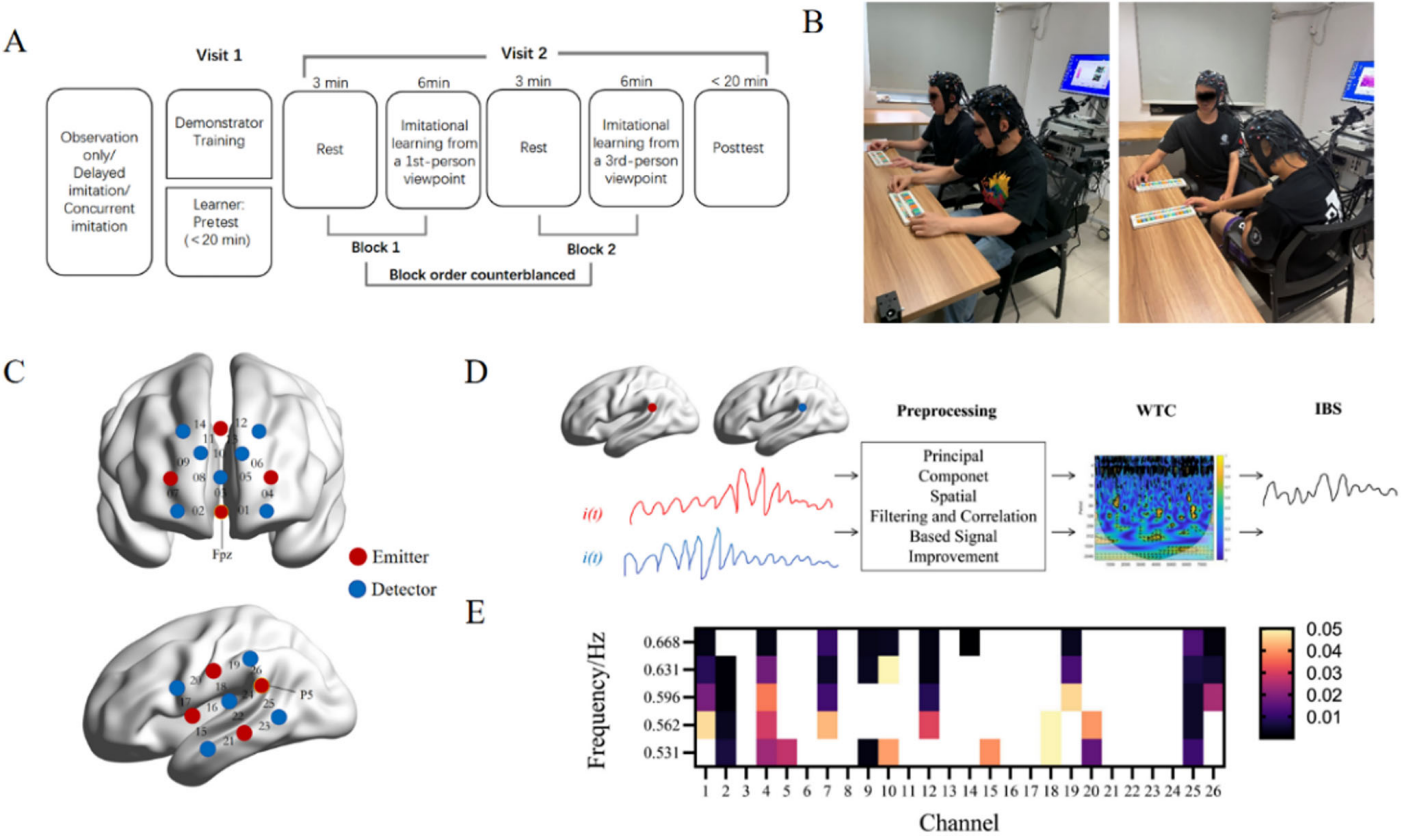
Experiment procedure, optode probe locations, and overview of the IBS analysis. (A) During the first visit, the learner completed the pretest while the demonstrator received abacus training. During the second visit, each dyad participated in two observational learning blocks (order counterbalanced), each consisting of a 3‐min rest and a 6‐min learning phase. After the learning phase, the learner completed the posttest within 20 min. (B) During the experiment, the demonstrator and learner sat face to face or side by side. The learner learned abacus skills through the assigned observational learning strategy according to the experimental conditions. A camera was placed in the opposite direction. (C) Optode probes were placed over the prefrontal and frontoparietal cortices. (D) Overview of the IBS analysis. Channel‐wise raw time series data were extracted from both participants in each dyad. After preprocessing, IBS was estimated by wavelet transform coherence (WTC) between the two clean signals (i, j: participants; t: time). (E) The 0.53–0.67 Hz frequency band was identified as the frequency of interest to compute time‐averaged IBS through a series of analyses.

Learners and instructors visited the laboratory twice over 2 days (Figure 1A). During their first visit, learners completed a pretest in 20 min. Instructors received 1 h of training in abacus addition and subtraction (see Table 1) and on delivering the demonstration. Following instructions from an experimenter, instructors watched a demonstration video that showed each step of the abacus procedure, accompanied by oral explanations based on the corresponding rhymes. They then studied modeling scripts that specified the goals, key difficulties, and procedural steps to ensure consistency across all instructors. Instructors were required to practice until they could demonstrate the abacus skills accurately, fluently, and confidently.

The second visit involved the fNIRS hyperscanning experiment on the observational learning of abacus skills. Each instructor underwent a one‐to‐one proficiency check with an experimenter. Their demonstration was required to match the modeling procedure specified in the scripts, including the pace of the abacus operations and the accompanying oral commentary (Liu et al. 2019; Pan et al. 2020). Throughout the experiment, the instructor and learner wore the fNIRS device (NIRScout X) on their heads to record brain activity (Figure 1B), while a video camera captured their behavior.

The experimental procedure for each group involved different combinations of learning content and learner perspective. The combinations were: (i) abacus addition with the first‐person viewpoint in Block 1 and abacus subtraction with the third‐person viewpoint in Block 2; or (ii) abacus addition with third‐person viewpoint in Block 1 and abacus subtraction with the first‐person viewpoint in Block 2. The block order was counterbalanced, and there was a 180‐s interval between blocks. Each block included a 180‐s resting phase as baseline, followed by a 360‐s observational learning phase. During the resting phase, participants sat quietly with their eyes closed. The observational learning phase involved either observation‐only, concurrent imitation, or delayed imitation with 16 dyads in each condition. Observation‐only and concurrent imitation each lasted 6 min, whereas delayed imitation lasted 10 min to learn either abacus addition or abacus subtraction in this study.

After the experiment concluded, the fNIRS devices were removed from the participants' heads, and the posttest was administered to evaluate their abacus skills within 20 min. The entire posttest procedure was recorded on video for subsequent analysis of abacus performance. To maintain the experiment's internal validity of the experiment, participants also completed the Corsi block‐tapping task to assess the potential impact of spatial working memory on observational learning and outcomes.

### 2.5 Behavior Data Analysis

The pretest and posttest each included two short‐answer problems (10 points) assessing participants’ understanding of the decimal and quinary systems, and 16 abacus operation problems (56 points) measuring accuracy in executing abacus steps. The equivalence in difficulty between the pretest and posttest was confirmed through a pilot study involving five participants, yielding high consistency between the two tests (*r* = 0.924, *p* < 0.001).

Two raters who were blind to the experimental design evaluated the pretest and posttest of each participant. Because no participant answered any pretest items correctly, inter‐rater reliability for the posttest was assessed using the intraclass correlation coefficient (ICC) of scores: short‐answer problems (ICC = 0.97), abacus operation problems (ICC = 0.99), and the total score (ICC = 0.99). The final scores were obtained by averaging the ratings of the two raters.

### 2.6 FNIRS Data Acquisition and Preprocessing

The fNIRS device used was the NIRScout X to simultaneously measure brain activity of both the instructor and the learner during the experiment. Two optode probe sets were used for each participant (Figure 1C). One 4 × 7 optode probe was placed over the prefrontal cortex and frontoparietal network (four emitters and seven detectors, resulting in 14 measurement points with a 3 cm source‐detector separation), while another 4 × 5 optode probe was placed over the left temporoparietal cortex (four emitters and five detectors forming 12 measurement points). The probe placements were adjusted to ensure consistent positions across participants. The correspondence between the NIRS channels (CHs) and the measured points on the cerebral cortex was determined using the virtual registration approach (Singh et al. 2005; Tsuzuki et al. 2007) for detailed MNI coordinates. The brain areas measured were associated with information processing, particularly action observation and imitation between the instructor and the learner (Caspers et al. 2010; Heiser et al. 2003; Pan et al. 2020; Zhu et al. 2022). The optical data were collected using wavelengths of 780 and 830 nm with a sampling rate of 7.8 Hz.

The fNIRS data were preprocessed using custom MATLAB R2023b scripts and the Homer2 toolbox (version 2.8) (Huppert et al. 2009). Initially, the raw light‐intensity time series were converted to changes in optical density (OD), which were then filtered using a bandpass filter (0.01–1 Hz) to reduce slow drift and high‐frequency noise (Figure 1D). A wavelet‐based denoising method was applied to eliminate global physiological noise (Duan et al. 2018). The OD data were transformed into oxyhemoglobin (HbO) and deoxyhemoglobin (HbR) signals based on the modified Beer–Lambert law (Cope and Delpy 1988), and a correlation‐based signal improvement (CBSI) procedure was employed to mitigate motion artifacts resulting from head movements (Cui et al. 2010). HbO signals were chosen for WTC computation due to their superior signal‐to‐noise ratio compared to HbR (Goldstein et al. 2018; Mahmoudzadeh et al. 2013) and widespread usage in similar studies (e.g., Dai et al. 2018; Hu et al. 2017; Pan et al. 2020; Zhu et al. 2022). Following automated preprocessing steps, the data quality was assessed by visual inspection. The anatomical locations of the channels were estimated using the NIRS_SPM package in MATLAB, which mapped each channel to its corresponding Brodmann area.

### 2.7 FNIRS Data Analyses

*Task‐related IBS analysis*. In line with previous research (Pan et al. 2020; Zhu et al. 2022), we used the wavelet transform coherence (WTC) algorithm to estimate the IBS between two HbO signals from dyads at corresponding channels (Grinsted et al. 2004; Torrence and Compo 1998). This process resulted in a two‐dimensional WTC matrix.

$$WTC\left(t,s\right)=\frac{|\langle s^{-1}W^{ij}\left(t,s\right)\rangle {|}^{2}}{\left|\langle s^{-1}W^{i}\left(t,s\right)\rangle {|}^{2}\right|\langle s^{-1}W^{j}\left(t,s\right)\rangle {|}^{2}}$$

Where *t* denotes time, *s* denotes the wavelet scale, 〈·〉 represents a smoothing operation in time and scale, and W is the continuous wavelet transform.

Task‐related IBS for each dyad was calculated as IBS_task_ − IBS_rest_ and then transformed into *z* values. A one‐sample *t*‐test was conducted to examine whether the time‐averaged IBS of each dyad in each channel was significantly higher during the task than during rest for each frequency band (0.01−1 Hz) (Nozawa et al. 2016). A set of FDR‐corrected *p*‐values was obtained to identify the frequency band of interest (FOI) for further analysis.

We performed a permutation test to show that the increased brain coupling resulted from observational learning between participants within each dyad rather than from shared environmental noise. The data from 96 participants were reshuffled in a pseudorandom manner to create 48 new dyads (e.g., time series from Demonstrator 3 paired with those from Learner 5). The WTC algorithm was then used to calculate the IBS of the pseudo‐dyads. This procedure was repeated 1000 times. An independent *t*‐test was used to compare the average IBS between the original dyads and the pseudo‐dyads with 1000 permutations.

We calculated the average IBS values within each FOI. Then, we determined the task‐related IBS values by subtracting resting‐state IBS from IBS during the observational learning phase and converting them to *z* values using Fisher's *z* transformation (Figure 1E). Subsequently, we conducted a 3 × 2 mixed ANOVA to examine significant differences among conditions, with imitation type as the between‐subjects factor and learner perspective as the within‐subjects factor.

*IBS analysis during the observational learning phases*. To examine whether observation, imitation, or both impact observational learning outcomes, we averaged the IBS values across the channels of interest (CHs) that showed significant IBS. The brain‐to‐brain coupling time series from these CHs were then resampled to 1 Hz to match the video recordings.

An undergraduate majoring in applied psychology was recruited to segment the overall learning process into different phases. The delayed imitation process was divided into observation and imitation phases. The observation phase started when the instructors lifted their fingers to operate the abacus and ended when they returned the abacus to its original places. The imitation phase began when the learner reproduced the first step of the abacus operation and ended when all the abacuses were returned to their original places. After the learner completed the imitation, the demonstrator showed the next abacus operation. The learning process in the observation‐only condition was divided into multiple observation phases because the learners were not required to imitate after observation, and the demonstrators were asked to show the next abacus operation after the previous one had finished. In the concurrent imitation condition, the learning process was divided into the same phases as the observation‐only condition, since the learner observed and imitated the demonstrated actions simultaneously.

We examined IBS during different learning phases. Initially, we averaged IBS in observation phases across three types of observational learning under the conditions of the first‐person and the third‐person viewpoints, respectively, and compared IBS using a mixed‐design repeated‐measures ANOVA. Subsequently, we averaged IBS in the imitation phase of the delayed imitation condition, and compared it with IBS during the observation phase of the observation‐only and concurrent imitation learning conditions. This comparison was based on the observation–execution matching property of the mirror neuron system (Caspers et al. 2010; Krüger et al. 2014), which posits that imitation involves the reproduction of previously observed actions (Bhat et al. 2017; Meltzoff 1988). Lastly, we compared IBS in observation and imitation phases in the delayed imitation condition using a repeated‐measure ANOVA.

*Relationship between IBS and learning outcomes*. We used Pearson correlation analysis to explore whether IBS from the CHs across different experimental conditions was associated with total learning outcomes, abacus knowledge outcomes, and abacus operation outcomes.

## 3 Results

The one‐way ANOVA revealed no significant differences in learning interest (*F* (2, 45) = 0.148, *p* = 0.86) or spatial working memory (*F* (2, 45) = 0.873, *p* = 0.43) across the three conditions. This suggests that neither of these factors affected IBS or learning outcomes. The subsequent analyses examined the effects of imitation type and perspective on IBS and abacus learning outcomes.

### 3.1 Abacus Learning Outcomes Analysis

**FIGURE 2:**
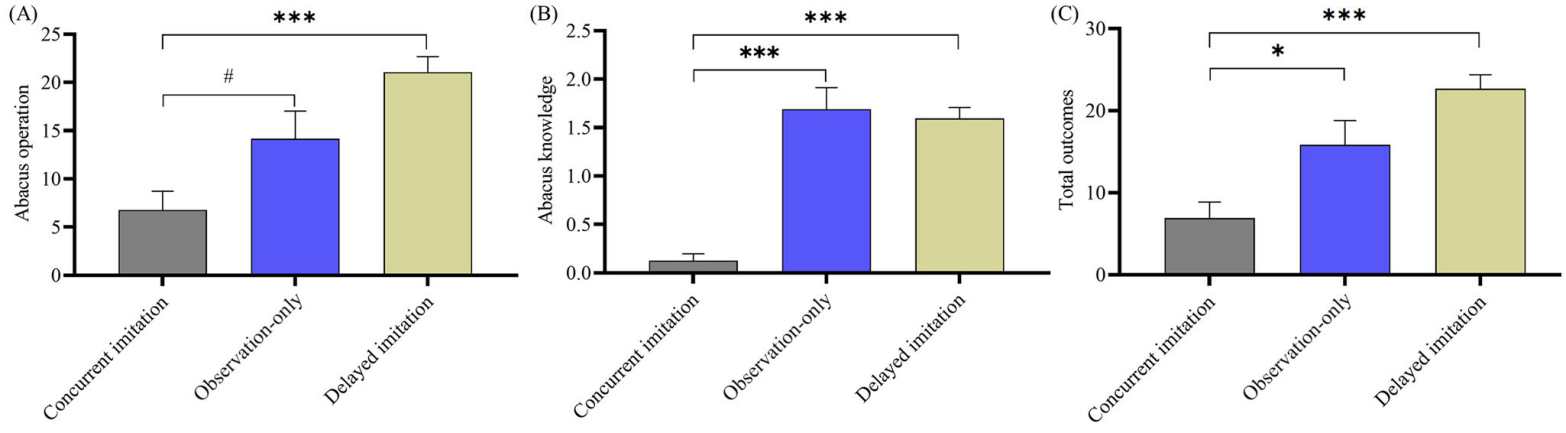
Learning outcomes among all conditions. (A) Participants in delayed imitation and mere observation‐only conditions showed better abacus operation performance than those in concurrent imitation condition. (B) Individuals in delayed imitation and mere observation‐only condition showed better abacus knowledge outcomes than those in concurrent imitation condition. (C) Individuals in the delayed imitation and mere observation‐only condition showed better overall abacus learning outcomes than those in concurrent imitation condition. Error bars represent standard errors of the mean (SEM). ***p < 0.001, **p < 0.01, *p < 0.05, ^#^ *p *< 0.01.

A repeated‐measure ANOVA was conducted to examine the effects of learner perspective (within‐subjects variable) and imitation type (between‐subjects variable) on abacus learning outcomes. The analysis of abacus operation performance revealed a significant main effect of imitation type (*F* (2, 45) = 10.415, *p* < 0.001, *η*_p_^2^ = 0.32). The results showed that delayed imitation led to higher outcomes than concurrent imitation (*p* < 0.001), and the mere observation‐only group showed marginally higher outcomes than the concurrent imitation groups (*p = *0.069). There were no differences between mere observation‐only and delayed imitation (*p* = 0.10; Figure 2A). In addition, the main effect of learner perspective (*F* (1, 45) = 1.66, *p* = 0.21) and the interaction effect between them (*F* (2, 45) = 0.06, *p* = 0.95) were not significant.

In addition, there was a significant main effect of imitation type on abacus knowledge outcomes: *F* (2, 45) = 33.926, *p* < 0.001, *η*_p_^2^ = 0.60. Both delayed imitation and mere observation‐only resulted in higher outcomes than concurrent imitation (*p*_s_ < 0.001). No difference was found between only observation‐only and delayed imitation (*p* > 0.999; Figure 2B). No main effect of learner perspective (*F* (1, 45) = 0.87, *p* = 0.37) or an interaction effect (*F* (2, 45) = 2.61, *p* = 0.084) on abacus knowledge outcomes was found.

Similarly, the results showed a significant main effect of imitation type on total learning outcomes (*F* (2, 45) = 12.181, *p* < 0.001, *η*_p_^2^ = 0.35). Post hoc analysis revealed that the delayed imitation group (*p* < 0.001) and mere observation‐only group (*p* = 0.023) showed better learning outcomes than the concurrent imitation group. There was no difference between the observation‐only group and the delayed imitation group (*p* = 0.12; Figure 2C). No significant main effect of learner perspective was found (*F* (1, 45) = 1.50, *p* = 0.23), nor was there a significant interaction effect (*F* (2, 45) = 0.01, *p = *0.995).

### 3.2 Brain Synchronization Related to Observation Learning

We investigated whether observational learning elicited higher IBS than the resting state using a permutation test. We compared the WTC values between the learning phase and the resting state using paired *t*‐tests. The results revealed a concentrated cluster of higher coherence values during the learning phase within the frequency range of 0.53–0.67 Hz. Therefore, we selected this frequency band for all subsequent analyses. This frequency range avoids overlap with the typical fundamental frequencies of respiratory (∼0.15–0.30 Hz) and cardiac (∼0.8–2.5 Hz) activities. It also aligns with previous fNIRS hyperscanning research demonstrating functional IBS in the 0.5–0.7 Hz band (Zheng et al. 2018). We defined seven regions of interest (ROIs) based on the spatial distribution of the measurement channels. These regions included the left medial prefrontal cortex (mPFC; Channels 3, 10), left dorsolateral prefrontal cortex (dlPFC) (left dlPFC; Channels 6, 12, 13), right dlPFC cortex (right dlPFC; Channels 9, 11, 14), left orbitofrontal (OFC) cortex and frontopolar (FPA) area (left OFC/FPA; Channels 1, 4, 5), right OFC cortex and FPA area (right OFC/FPA; Channels 2, 7, 8), left sensorimotor cortex (left SMC; Channels 15–20), and left temporoparietal area (left TP; Channels 21–26).

**FIGURE 3:**
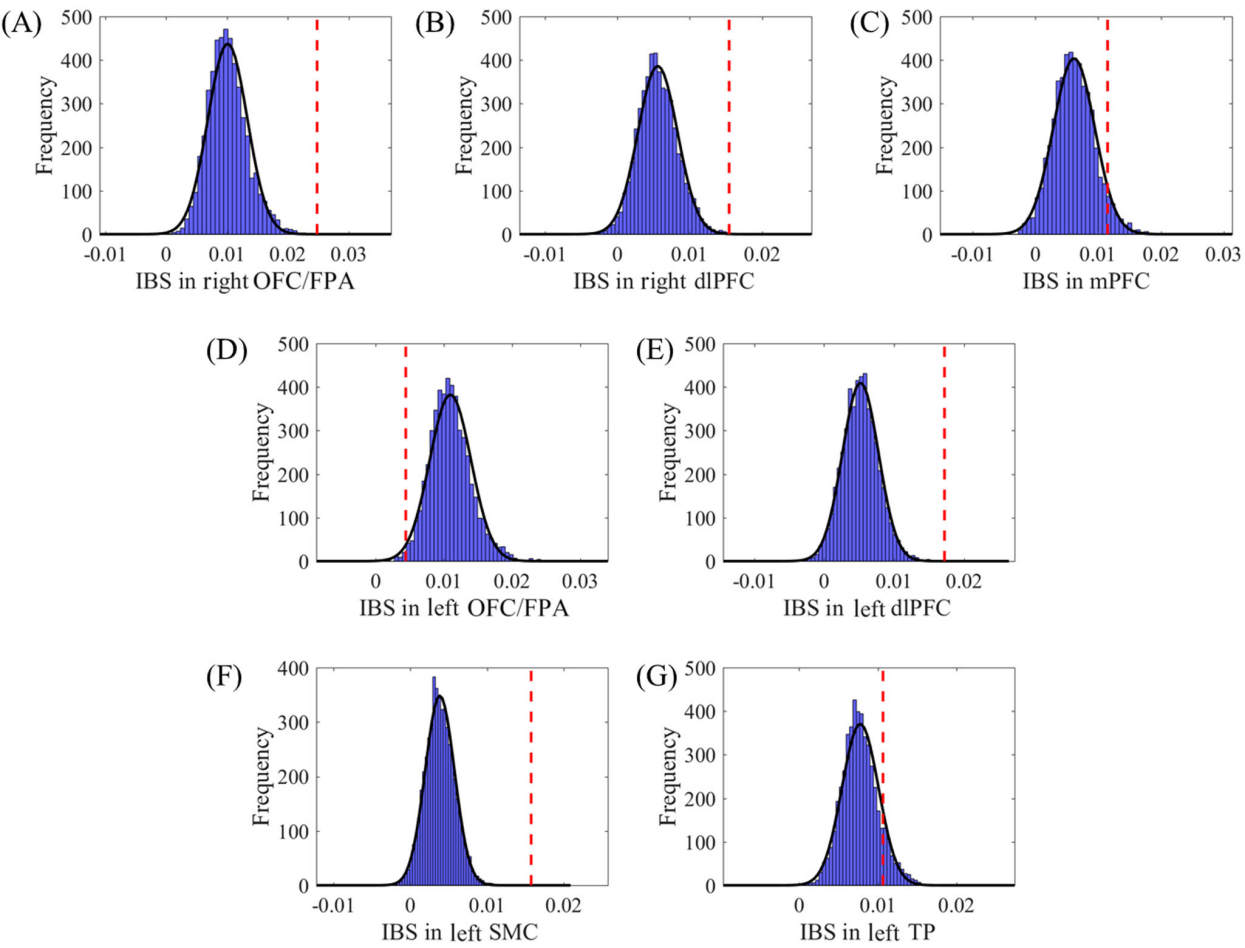
Results of the ROI‐based permutation analysis. The permutation analysis showed that the enhanced brain‐to‐brain couplings were shown in (A) right OFC/FPA, (B) right dlPFC, (E) left dlPFC, and (F) left SMC. In contrast, true dyads did not exhibit significantly higher IBS than pseudo‐dyads in the (C) mPFC, (D) left OFC/FPA, and (G) left TP.

To confirm that the observed IBS was driven by genuine social interaction rather than shared environmental factors or common sensory inputs, we conducted an ROI‐based permutation test with 5000 iterations. We compared the mean IBS in the true dyads against a null distribution generated from randomly paired pseudo‐dyads. The permutation analysis revealed that true dyads exhibited significantly higher IBS than pseudo‐dyads in four ROIs: right OFC/FPA (*p* < 0.001; Figure 3A), right dlPFC (*p* = 0.001; Figure 3B), the left dlPFC (*p* < 0.001; Figure 3E), and left SMC (*p* < 0.001; Figure 3F). In contrast, no significant differences were observed in the mPFC (*p* = 0.08; Figure 3C), left OFC/FPA (*p* = 0.99; Figure 3D), and left TP (*p* = 0.11; Figure 3G). These findings are consistent with studies demonstrating that observational learning evoked higher IBS in the brain regions mentioned above during observational learning (Bhat et al. 2017; Kostorz et al. 2020; Pan et al. 2020; Su et al. 2020).

To further rule out the influence of broad physiological noise, we conducted a control analysis of adjacent frequency bands (0.15–0.30, 0.40–0.53, and 0.67–0.80 Hz). This analysis revealed no significant IBS effects within these adjacent bands (Figure S1). These results indicate that observational learning elicited increased brain‐to‐brain coupling primarily within the bilateral dlPFC, right OFC/FPA, and left sensorimotor regions (SMC).

### 3.3 The Interpersonal Brain Synchronization Under Different Conditions

**FIGURE 4:**
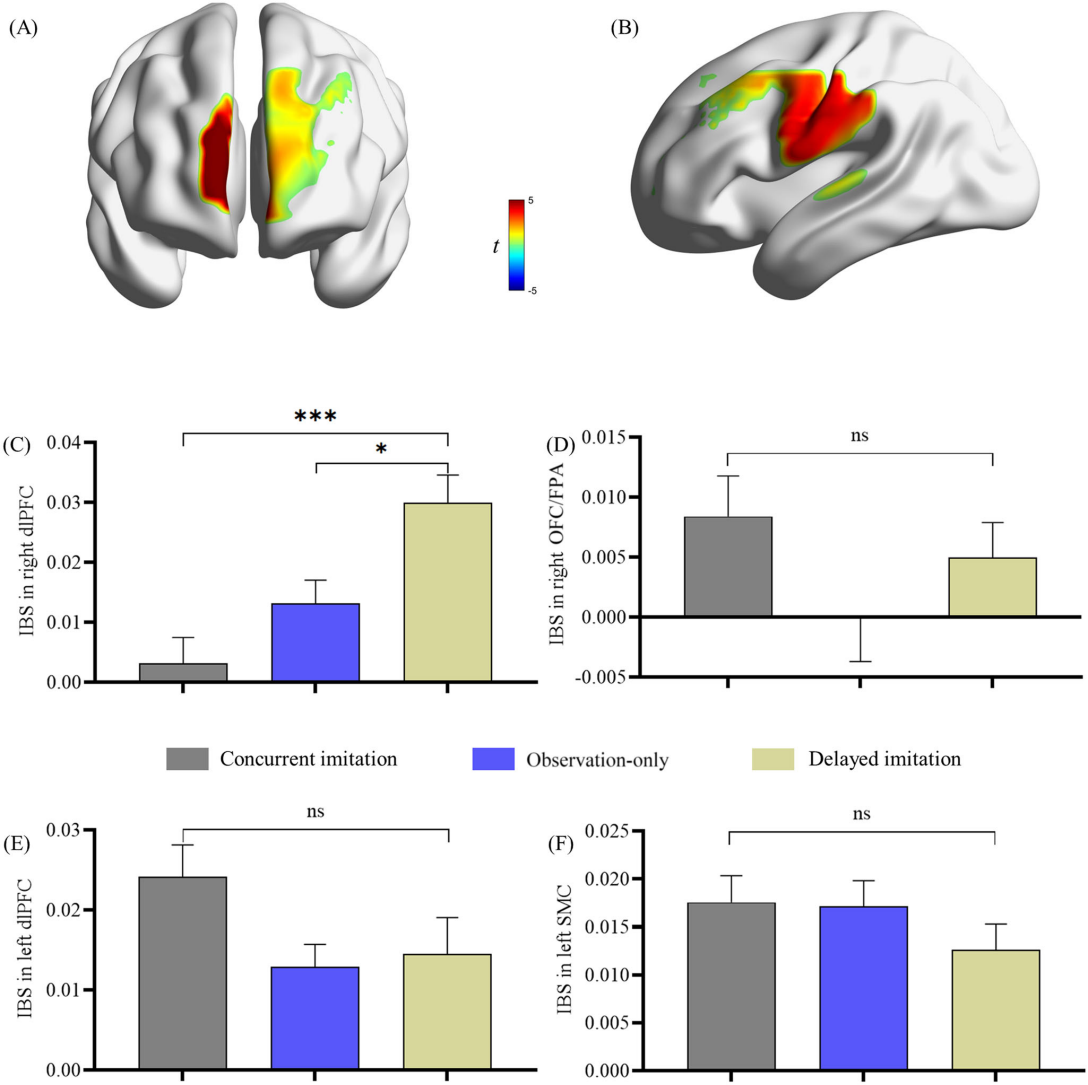
Interpersonal brain synchronization across imitation types. (A, B) Brain maps displaying the *t*‐values for all participants during the learning phase compared to the resting state. (C) In the right dlPFC, participants in the delayed imitation group exhibited significantly higher IBS compared to both the concurrent imitation and mere observation‐only groups. (D–F) No significant differences in IBS were observed among the three groups in the (D) right OFC/FPA, (E) left dlPFC, and (F) left SMC. *Note*: *p < 0.05, ***P < 0.001, ns p < 0.05.

We further examined differences in IBS across conditions, and focusing on the four ROIs that showed significant IBS in the permutation test. The repeated‐measures ANOVA was conducted to examine IBS with learner perspective as a within‐subjects factor and imitation type as a between‐subjects factor (Figure 4A,B). The results showed a significant main effect of imitation type on IBS in the right dlPFC: *F* (2, 45) = 9.991, FDR‐corrected *p* = 0.001, *η*_p_^2^ = 0.31. Participants in the delayed imitation group showed higher IBS than those in the concurrent imitation group (*p* < 0.001) and the observation‐only group (*p* = 0.024), but the difference between the observation‐only and concurrent imitation groups was not significant (*p* = 0.32; Figure 4C). The main effect of perspective (*F* (1, 45) = 1.922, FDR‐corrected *p* = 0.35) and the interaction between learner perspective and group (*F* (2, 45) = 1.182, FDR‐corrected *p* = 0.63) were not significant.

However, for the right OFC/FPA (Figure 4D), left dlPFC (Figure 4E), and left SMC (Figure 4F), the analysis did not yield any significant main or interaction effects on IBS (*F < *2.485, FDR‐corrected *p*_s_ > 0.095).

### 3.4 Interbrain Synchronization During Different Phases

As an exploratory analysis, to understand why delayed imitation elicited higher IBS, we compared IBS in specific phases of observational learning among different imitation types. Video coding was conducted for each dyad. The delayed imitation recordings were divided into observation and imitation phases, while the observation‐only recordings and the concurrent imitation recordings were divided into a series of observation phases because learners did not perform a separate imitation phase. The time courses of brain‐to‐brain coupling were aligned with video‐coded behavior by downsampling to 1 Hz. IBS was extracted from each observational learning phase and averaged within each condition. Task‐related IBS was then obtained by subtracting brain‐to‐brain coupling during the resting phase from the averaged coupling segments during different phases of observational learning.

**FIGURE 5:**
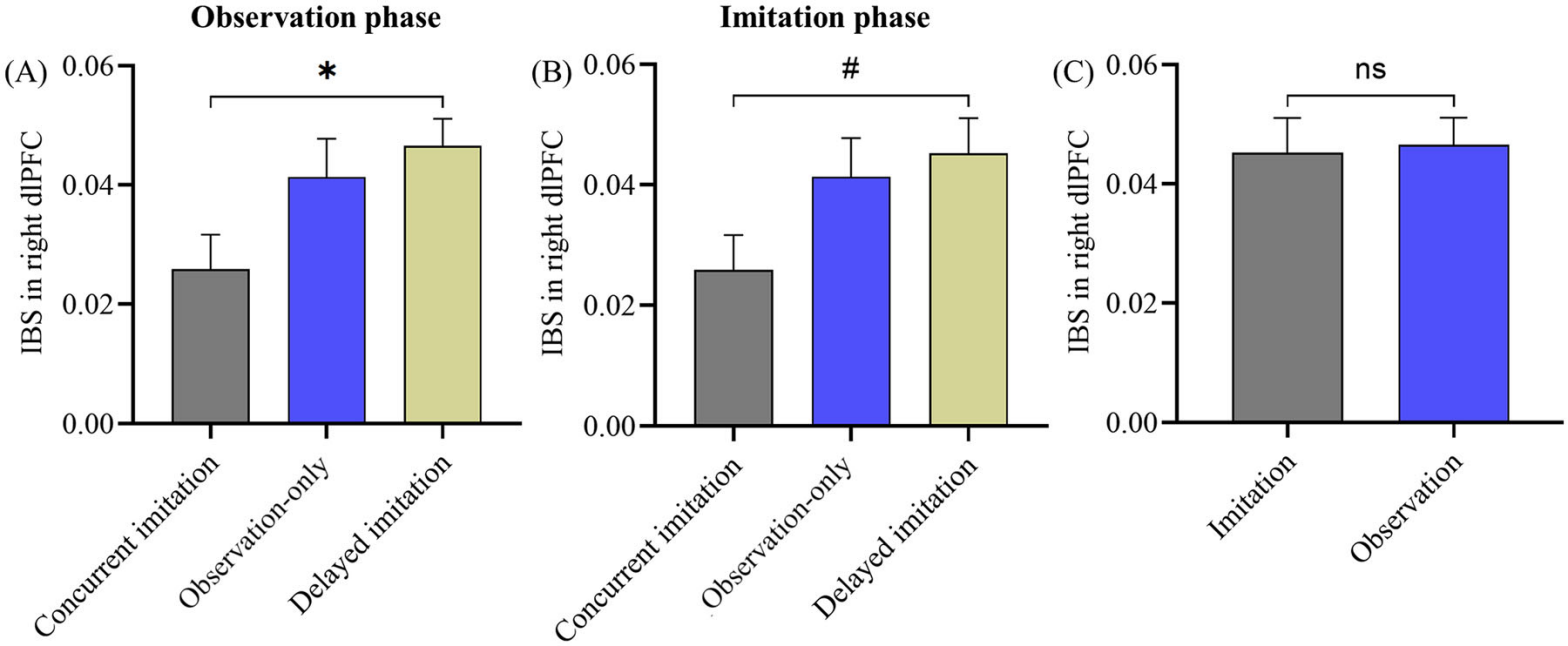
Comparison of IBS during observation and imitation phases across three types of observational learning. (A) In the right dlPFC, participants in the delayed imitation group showed significantly higher IBS than those in the concurrent imitation group during the observation phase. (B) Participants in the delayed imitation group showed marginally higher IBS than those in the concurrent imitation group during the imitation phase. (C) There was no significant IBS difference between the observation phase and the imitation phase in the right dlPFC. *Note*: *p <0.05, # 0.05 < p < 0.01, ns p > 0.05.

Because no significant effect of learner perspective was found, we compared the average IBS during the observation phases across the three conditions. Furthermore, because the right dlPFC was the only region that exhibited a significant difference in IBS among the learning conditions, we focused our subsequent phase‐specific analyses on this region. First, we compared IBS in the observation phase. The results showed a significant main effect of imitation type in the right dlPFC (*F* (2, 45) = 3.621, *p* = 0.035, *η*_p_^2^ = 0.14; Figure 5A). Participants in the delayed imitation group showed higher IBS than those in the concurrent imitation group (*p* = 0.039), but the difference between the observation‐only group and the concurrent imitation group was not significant (*p* > 0.999).

Second, IBS in the imitation phase in the delayed imitation condition was compared with that in the observation phases of the mere observation‐only and concurrent imitation conditions. The results also showed a marginally significant difference among the three conditions in the right dlPFC (*F* (2, 45) = 2.863, *p* = 0.068, *η*_p_^2^ = 0.11; Figure 5B). Participants in the delayed imitation group showed higher IBS than those in the concurrent imitation group (*p* = 0.086), but the difference between the observation‐only group and the concurrent imitation group was not significant (*p* > 0.999).

Finally, a paired *t*‐test was conducted to compare the average IBS during the observation and imitation phases. No significant difference in IBS was found between these two phases in the right dlPFC (*t* (15) = 0.475, *p* = 0.64; Figure 5C).

These findings suggest that both observation and imitation behaviors contribute to the brain‐to‐brain coupling in delayed imitation.

### 3.5 Correlation Between IBS and Learning Outcomes

**FIGURE 6:**
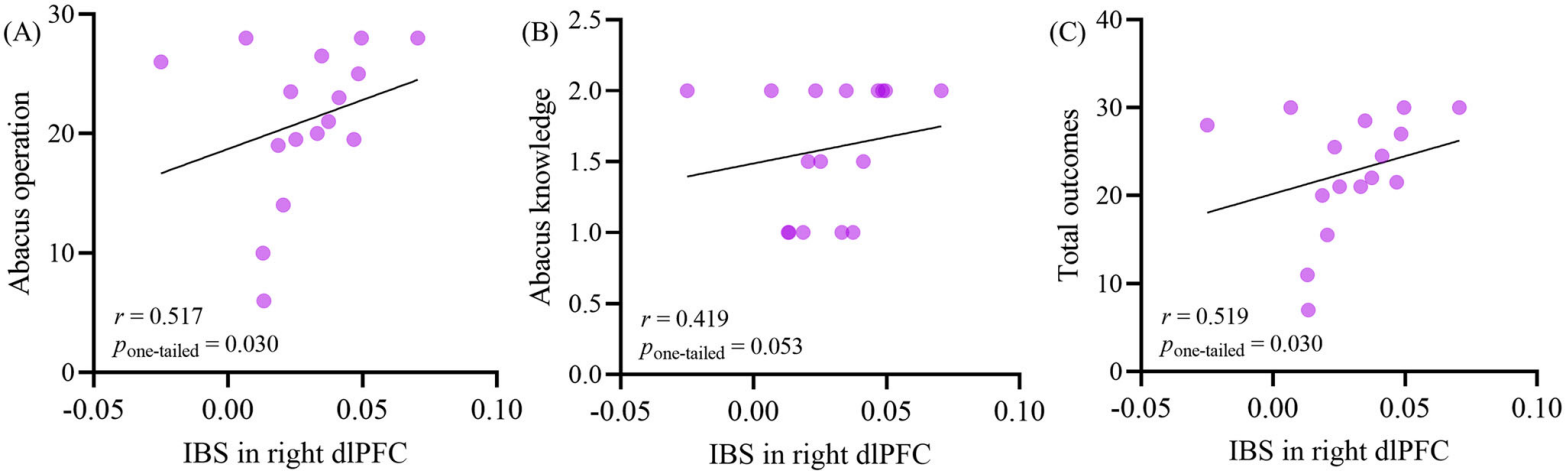
Correlations between interpersonal brain synchronization in the right dlPFC and learning outcomes. The plots illustrate significant positive relationships between synchronization in the right dlPFC and (A) abacus operation, (B) abacus knowledge, and (C) total scores.

To investigate the relationship between IBS and learning outcomes, we conducted a Pearson correlation analysis. In the delayed imitation group (*n* = 16), IBS in the right dlPFC was positively associated with learning outcomes. Specifically, greater right dlPFC IBS was significantly correlated with better abacus operation performance (*r* = 0.517, FDR‐corrected one‐tailed *p* = 0.030, 95% CI [0.029, 0.806]; Figure 6A) and better total outcomes (*r* = 0.519, FDR‐corrected one‐tailed *p* = 0.030, 95% CI [0.032, 0.807]; Figure 6C). In addition, there was a marginally significant positive correlation between right dlPFC IBS and abacus knowledge (*r* = 0.419, FDR‐corrected one‐tailed *p* = 0.053, 95% CI [−0.097, 0.757]; Figure 6B). In contrast, no significant relationship was found between abacus learning outcomes and IBS in either the concurrent imitation and mere observational‐only learning conditions (FDR‐corrected *p*_s_ > 0.48).

## 4 Discussion

This study investigated the impact of imitation strategies on observational learning and the associated neural basis. Behaviorally, learners in the delayed imitation group outperformed those in the concurrent imitation group in total learning outcomes, abacus knowledge, and operational skills. Furthermore, the results revealed that delayed imitation elicited stronger IBS in the right dlPFC compared with the concurrent imitation condition. Further analyses showed that both observation and imitation stages contributed to increased IBS during delayed imitation. Importantly, we found that IBS in the right dlPFC during delayed imitation was positively correlated with abacus learning outcomes. These findings highlight the effectiveness of delayed imitation, suggesting that enhanced interbrain coupling between instructor and learner may facilitate learning outcomes. This study provides new insights into the interpersonal neuroscience mechanisms supporting effective observational learning.

### 4.1 Delayed Imitation Improves Abacus Learning Outcomes More Than Concurrent Imitation

In this study, delayed imitation learning led to higher learning outcomes for abacus knowledge, operations, and overall outcomes than concurrent imitation learning. This finding aligns with previous studies by Crone et al. (2021) and Weeks et al. (1996). In concurrent imitation learning, individuals had to observe and imitate the demonstrator's actions simultaneously, leading to shallower processing of sensorymotor information and reduced retention of abacus operations and rules. On the other hand, individuals in delayed imitation condition did not have to reproduce the demonstration immediately, allowing them to process and retain abacus operations more deeply, thereby enabling better reenactment later (Crone et al. 2021; Weeks et al. 1996). There was no significant difference in learning outcomes between delayed imitation and observation‐only learning, which is consistent with some previous studies (Fiorella et al. 2017; Sweller et al. 2020). This finding may be attributed to both groups intentionally observing the demonstration behaviors (Sweller et al. 2020). Moreover, in line with embodied cognition theory and the literature on mirror neuron systems, individuals in observational‐only learning condition can mentally simulate the demonstration, leading to implicit learning (Barsalou 2008; Rizzolatti and Craighero 2004). However, this study's results diverge from other studies (Blandin et al. 1999; Cherdieu et al. 2017; Deakin and Proteau 2000; Zhidan, Williamson, et al. 2015). Previous studies have shown that delayed imitation can improve motor behavior performance better than only observation‐only (Blandin et al. 1999; Shea et al. 2000), especially for children (Zhidan, Williamson, et al. 2015). For example, Zhidan, Williamson, et al. (2015) found that delayed imitation helped young children learn complex concepts, such as weight. The discrepancy between these previous findings and our current results may stem from differences in task difficulty. Sorting identical objects based on weight is challenging for children; imitating adult hefting behavior, and combines bodily experience and cognition into a cognitive schema facilitates a better understanding of weight concepts (Zhidan, Williamson, et al. 2015). In contrast, basic abacus addition and subtraction skills are relatively simple tasks for undergraduate students. Because the task was not highly demanding, participants could likely comprehend and acquire the skills through observation alone, reducing the potential additive benefits of delayed imitation. Another possible factor is the timing of assessment. We evaluated learning outcomes immediately after observational learning. Cherdieu et al. (2017) reported no significant difference in immediate learning outcomes between delayed imitation and observation‐only alone, but found that delayed imitation led to superior delayed learning outcomes. This finding suggests that delayed imitation might primarily enhance memory and learning over a longer period by adding motor behaviors as retrieval cues. Given the conflicting results, future research should investigate how task complexity and testing intervals influence the relationship between imitation strategies and learning outcomes.

More importantly, previous studies in educational psychology have predominantly examined the impact of the delayed imitation strategy on motor skills by assessing the accuracy and speed of motor actions (Crone et al. 2021; Fiorella et al. 2017; Sweller et al. 2020). This study, however, found that delayed imitation not only improved performance in abacus operations but also enhanced understanding of the underlying abacus rules. According to embodied cognition theory (Barsalou 2008, 2010), action execution influences cognitive processes such as memory and understanding. For example, research has shown that gesturing, which is akin to imitation, during mathematical tasks can enhance the comprehension of mathematical concepts and strategies (Broaders et al. 2007; Cook and Goldin‐Meadow 2006; Cook et al. 2008). Thus, this study expands the scope of the benefits of delayed imitation from motor skills to intellectual skills.

The observer's viewpoint did not impact abacus learning outcomes in this study. The findings of this study align with those of Boucheix et al. (2018) and Leng et al. (2024). However, these findings differ from previous studies, which found that the first‐person viewpoint led to better learning outcomes than the third‐person viewpoint due to greater mental transformation demands (de Koning et al. 2023; Fiorella et al. 2017; Garland and Sanchez 2013). A possible explanation lies in the relative simplicity of the abacus operations. There are only two directions in an abacus operation: either upward or downward movements along the columns of the abacus, eliminating the need for mental transformation when using the third‐person viewpoint. Future studies are needed to explore the effect of viewpoint on learning.

### 4.2 The Increased IBS in Delayed Imitation Learning Is Associated With Abacus Learning Outcomes

To our knowledge, this study is the first to investigate the relationship between observational learning strategies and IBS. We found that delayed imitation learning elicited significantly higher IBS in the right dlPFC than both observation‐only and concurrent imitation learning. No significant differences in IBS were observed in the parietal cortex across the three conditions, which differs from findings reported by Pan et al. (2023) and Zhu et al. (2022). This may be because the abacus demonstrations provided by the demonstrator were identical across conditions. Because all learners were exposed to the same visual input, they did not exhibit significant differences in the perceptual and integrative processing of the observed actions, resulting in comparable levels of IBS within the parietal cortex. By contrast, the dlPFC, which is responsible for working memory, action monitoring, and executive regulation, is more responsive to changes in imitation strategies. This responsiveness led to the condition‐dependent differences in IBS observed in this study.

The task structure and timing cues of delayed imitation impose unique cognitive demands compared with observation‐only or concurrent imitation. Delayed imitation necessitates deeper prediction and decoding of demonstrated actions during observation, along with monitoring and regulating reproduced actions during action execution. As a result, delayed imitation elicits notably higher activation in the right dlPFC than observational‐only or concurrent imitation learning. This finding is consistent with previous fNIRS hyperscanning studies, which have demonstrated the involvement of the prefrontal cortex in social cognitive learning, particularly in teacher–student interactions (Liu et al. 2019; Pan et al. 2020), and with single‐brain studies highlighting the role of the right dlPFC in imitation learning (Higuchi et al. 2012; Vogt et al. 2007). It is worth noting that the sequential characteristics of delayed imitation may also contribute to the increased IBS in the right dlPFC. While the permutation analysis mitigates concerns about shared environmental factors, it does not fully eliminate alternative explanations. Shared sensory input and task‐related entrainment, where both the demonstrator and learner attend to the same visual stimuli and synchronize their neural activity with the same external timeline, may independently contribute to neural alignment between participants. Although the current data do not definitively rule out these sensory and structural factors, the unique cognitive demands of delayed imitation provide the most straightforward explanation for the observed IBS advantage. In summary, the right dlPFC plays a crucial role in delayed imitation learning, which may support the benefits of this learning strategy for comprehending demonstrated actions and the underlying rules (Crone et al. 2021; Weeks et al. 1996).

Prior research on observational learning has often focused on either the observation phase (Crone et al. 2021; Fiorella et al. 2017) or the imitation phase (Meltzoff and Williamson 2013; Zhidan, Williamson, et al. 2015) separately. Our phase analysis revealed that delayed imitation elicited higher IBS than concurrent imitation during both the observation stage and the imitation stage, yet no significant difference in IBS was found between these two stages within the delayed imitation condition. This pattern suggests that the increased IBS in delayed imitation learning emerges from the learning episode as a whole rather than from individual sub‐phases. This interpretation is consistent with the role of the right dlPFC during both stages. It is involved in movement planning during the observation phase (Burke et al. 2010) and movement inhibition and error monitoring during the imitation phase (Higuchi et al. 2012), continuously monitoring whether reproduced actions are consistent with demonstrated actions. These findings, together with those of Higuchi et al. (2012) and Vogt et al. (2007), highlight the combined impact of observation and imitation in delayed imitation learning, supporting the theoretical accounts of Bandura (1977) and Meltzoff (2005, 2007). Consequently, the neural coupling between participants is likely sustained throughout the entire learning sequence.

In addition, IBS in the right dlPFC was positively correlated with abacus learning outcomes, including both abacus knowledge and operations. In the context of delayed imitation learning, the right dlPFC supports not only the perceptual encoding of demonstrated actions but also the retrieval and integration of visual and auditory information during observation, as well as monitoring imitation accuracy during execution, which is directly relevant to understanding abacus operations and their underlying rules (Burgess et al. 2000; Abujelala et al. 2021). Based on this correlation between right dlPFC interbrain coupling and abacus learning outcomes, together with prior evidence linking dlPFC activity to imitation‐based learning, our study suggests that right dlPFC interbrain coupling may support successful delayed imitation learning.

### 4.3 Limitations

Some limitations should be acknowledged in this study. First, the abacus addition and subtraction skills chosen for this study are considered simple for undergraduate participants. Furthermore, we evaluated learning outcomes immediately after the learning phase without assessing delayed outcomes. These factors may explain why delayed imitation learning did not show significant differences in outcomes and IBS compared with observation‐only learning. Future studies should use more challenging skills and assess delayed outcomes to better understand the differences among observation‐only, delayed imitation, and concurrent imitation strategies.

Second, this study focused solely on investigating IBS in the prefrontal cortex and left temporoparietal junction (TPJ), which is consistent with previous studies (Caspers et al. 2010; Heiser et al. 2003; Pan et al. 2020), due to the limited number of fNIRS channels. Activation of the left TPJ during observational learning has also been documented in prior literature (Sowden and Catmur 2015). Future fNIRS studies should compare IBS in the left TPJ across the three types of observational learning examined in the present study to identify additional brain regions associated with delayed imitation learning.

Third, 96 undergraduates were enrolled in this study based on previous studies (Pan et al. 2020; Zhu et al. 2022) due to the challenges of estimating the required number of participants using G*Power. Future studies should consider increasing the sample size to further elucidate the cognitive‐neural mechanisms underlying delayed imitation learning. In addition, it is noteworthy that a higher proportion of male undergraduates participated in this study, limiting the generalizability of the findings beyond male populations. Future studies should recruit more female participants to validate the findings of the present study.

## 5 Conclusion

This study demonstrates that delayed imitation learning enhances skill acquisition outcomes, specifically skill operation performance and comprehension of underlying knowledge, compared with concurrent imitation learning. However, under the current task conditions, delayed imitation does not provide an additional behavioral advantage over observation‐only learning. Unlike previous cognitive neuroscience studies that relied on video‐based experimental paradigms, this research employed a controlled experimental design combined with ecologically valid observations during imitation tasks to examine IBS between demonstrators and learners, as well as the resulting learning outcomes. The findings reveal heightened IBS in the right dlPFC during delayed imitation learning, reflecting the combined influence of observation and imitation. Moreover, IBS in the right dlPFC is associated with abacus skill learning outcomes, encompassing both operational performance and knowledge understanding. The concept of interbrain synchrony offers a novel perspective for understanding how delayed imitation influences observational learning outcomes and helps identify effective observational learning strategies.

## Author Contributions

C.Y. wrote the original and final manuscript. L.Y. wrote the final manuscript and analyzed the data. L.M. collected and analyzed the data. M.Y. collected the data. Y.H. read and approved the final manuscript.

## Funding

This work was supported by the Humanities and Social Sciences Foundation of the Ministry of Education of China (Grant No. 23YJAZH173).

## Ethics Statement

This work was approved by the Institutional Review Board of Jiangsu Normal University (JSNU‐CAIAS‐2024‐0428). All procedures were conducted in accordance with the Declaration of Helsinki, and written informed consent was obtained from each participant.

## Conflicts of Interest

The authors declare no conflicts of interest.

## Supporting information




**Supporting Material**: brb371683‐sup‐0001‐FigureS1.docx

## Data Availability

The datasets used and analyzed during the current study are available from the corresponding author upon reasonable request.

## References

[brb371683-bib-0071] Abujelala, M. , R. Karthikeyan , O. Tyagi , J. Du , and R. K. Mehta . 2021. “Brain Activity‐Based Metrics for Assessing Learning States in VR Under Stress Among Firefighters: an Explorative Machine Learning Approach in Neuroergonomics.” Brain Sciences 11, no. 7: 885. 10.3390/brainsci11070885. 34209388 PMC8304323

[brb371683-bib-0001] Bandura, A. 1977. Social Learning Theory. Prentice‐Hall.

[brb371683-bib-0002] Barhorst‐Cates, E. M. , M. W. Isaacs , L. J. Buxbaum , and A. L. Wong . 2022. “Does Spatial Perspective in Virtual Reality Affect Imitation Accuracy in Stroke Patients?.” Frontiers in Virtual Reality 3: 934642. 10.3389/frvir.2022.934642. 37063476 PMC10104493

[brb371683-bib-0003] Barsalou, L. W. 2008. “Grounded Cognition.” Annual Review of Psychology 59: 617–645. 10.1146/annurev.psych.59.103006.093639. 17705682

[brb371683-bib-0004] Barsalou, L. W. 2010. “Grounded Cognition: Past, Present, and Future.” Topics in Cognitive Science 2, no. 4: 716–724. 10.1111/j.1756-8765.2010.01115.x. 25164052

[brb371683-bib-0005] Bhat, A. N. , M. D. Hoffman , S. L. Trost , et al. 2017. “Cortical Activation During Action Observation, Action Execution, and Interpersonal Synchrony in Adults: A Functional Near‐Infrared Spectroscopy (fNIRS) Study.” Frontiers in Human Neuroscience 11: 431. 10.3389/fnhum.2017.00431. 28928646 PMC5591977

[brb371683-bib-0006] Blandin, Y. , L. Lhuisset , and L. Proteau . 1999. “Cognitive Processes Underlying Observational Learning of Motor Skills.” Quarterly Journal of Experimental Psychology Section A 52, no. 4: 957–979. 10.1080/027249899390882.

[brb371683-bib-0007] Boucheix, J.‐M. , P. Gauthier , J.‐B. Fontaine , and S. Jaffeux . 2018. “Mixed Camera Viewpoints Improve Learning Medical Hand Procedure From Video in Nurse Training?” Computers in Human Behavior 89: 418–429. 10.1016/j.chb.2018.01.017.

[brb371683-bib-0008] Broaders, S. C. , S. W. Cook , Z. Mitchell , and S. Goldin‐Meadow . 2007. “Making Children Gesture Brings Out Implicit Knowledge and Leads to Learning.” Journal of Experimental Psychology: General 136, no. 4: 539–550. 10.1037/0096-3445.136.4.539. 17999569

[brb371683-bib-0070] Burgess, P. W. , E. Veitch , A. de Lacy Costello , and T. Shallice . 2000. “The Cognitive and Neuroanatomical Correlates of Multitasking.” Neuropsychologia 38, no. 6: 848–863. 10.1016/S0028-3932(99)00134-7. 10689059

[brb371683-bib-0009] Caspers, S. , K. Zilles , A. R. Laird , and S. B. Eickhoff . 2010. “ALE Meta‐Analysis of Action Observation and Imitation in the Human Brain.” Neuroimage 50, no. 3: 1148–1167. 10.1016/j.neuroimage.2009.12.112. 20056149 PMC4981639

[brb371683-bib-0010] Cherdieu, M. , O. Palombi , S. Gerber , J. Troccaz , and A. Rochet‐Capellan . 2017. “Make Gestures to Learn: Reproducing Gestures Improves the Learning of Anatomical Knowledge More Than Just Seeing Gestures.” Frontiers in Psychology 8: 1689. 10.3389/fpsyg.2017.01689. 29062287 PMC5640824

[brb371683-bib-0011] Cook, S. W. , and S. Goldin‐Meadow . 2006. “The Role of Gesture in Learning: Do Children Use Their Hands to Change Their Minds?” Journal of Cognition and Development 7, no. 2: 211–232. 10.1207/s15327647jcd0702_4.

[brb371683-bib-0012] Cook, S. W. , Z. Mitchell , and S. Goldin‐Meadow . 2008. “Gesturing Makes Learning Last.” Cognition 106, no. 2: 1047–1058. 10.1016/j.cognition.2007.04.010. 17560971 PMC2265003

[brb371683-bib-0013] Cope, M. , and D. T. Delpy . 1988. “System for Long‐Term Measurement of Cerebral Blood and Tissue Oxygenation on Newborn Infants by Near Infra‐Red Transillumination.” Medical & Biological Engineering & Computing 26, no. 3: 289–294. 10.1007/BF02447083. 2855531

[brb371683-bib-0014] Crone, C. L. , L. M. Rigoli , G. Patil , et al. 2021. “Synchronous vs. Non‐Synchronous Imitation: Using Dance to Explore Interpersonal Coordination During Observational Learning.” Human Movement Science 76: 102776. 10.1016/j.humov.2021.102776. 33639354

[brb371683-bib-0015] Cui, X. , S. Bray , and A. L. Reiss . 2010. “Functional Near Infrared Spectroscopy (NIRS) Signal Improvement Based on Negative Correlation Between Oxygenated and Deoxygenated Hemoglobin Dynamics.” Neuroimage 49, no. 4: 3039–3046. 10.1016/j.neuroimage.2009.11.050. 19945536 PMC2818571

[brb371683-bib-0016] Dai, B. , C. Chen , Y. Long , et al. 2018. “Neural Mechanisms for Selectively Tuning in to the Target Speaker in a Naturalistic Noisy Situation.” Nature Communications 9, no. 1: 2405. 10.1038/s41467-018-04819-z. PMC6008393 29921937

[brb371683-bib-0017] Deakin, J. M. , and L. Proteau . 2000. “The Role of Scheduling in Learning Through Observation.” Journal of Motor Behavior 1, no. 32: 27–36. 10.1080/00222890009601377 10975274

[brb371683-bib-0018] de Koning, B. B. , K. Mok , N. Marcus , and P. Ayres . 2023. “Investigating the Role of Hand Perspective in Learning From Procedural Animations.” British Journal of Educational Psychology 93, no. S2: 251–269. 10.1111/bjep.12542. 36047932

[brb371683-bib-0019] Duan, L. , Z. Zhao , Y. Lin , X. Wu , Y. Luo , and P. Xu . 2018. “Wavelet‐Based Method for Removing Global Physiological Noise in Functional Near‐Infrared Spectroscopy.” Biomedical Optics Express 9, no. 8: 3805. 10.1364/BOE.9.003805. 30338157 PMC6191612

[brb371683-bib-0020] Fiorella, L. , T. van Gog , V. Hoogerheide , and R. E. Mayer . 2017. “It's All a Matter of Perspective: Viewing First‐Person Video Modeling Examples Promotes Learning of an Assembly Task.” Journal of Educational Psychology 109, no. 5: 653–665. 10.1037/edu0000161.

[brb371683-bib-0021] Francisco, V. , A. Decatoire , and C. Bidet‐Ildei . 2023. “Action Observation and Motor Learning: The Role of Action Observation in Learning Judo Techniques.” European Journal of Sport Science 23, no. 3: 319–329. 10.1080/17461391.2022.2036816. 35098899

[brb371683-bib-0022] Friedman, N. P. , and T. W. Robbins . 2022. “The Role of Prefrontal Cortex in Cognitive Control and Executive Function.” Neuropsychopharmacology 47, no. 1: 72–89. 10.1038/s41386-021-01132-0. 34408280 PMC8617292

[brb371683-bib-0066] Fronda, G. , and M. Balconi . 2020. “The Effect of Interbrain Synchronization in Gesture Observation: a fNIRS Study.” Brain and Behavior 10, no. 7: e01663. 10.1002/brb3.1663. 32469153 PMC7375069

[brb371683-bib-0023] Garland, T. B. , and C. A. Sanchez . 2013. “Rotational Perspective and Learning Procedural Tasks From Dynamic Media.” Computers & Education 69: 31–37. 10.1016/j.compedu.2013.06.014.

[brb371683-bib-0024] Goldstein, P. , I. Weissman‐Fogel , G. Dumas , and S. G. Shamay‐Tsoory . 2018. “Brain‐to‐Brain Coupling During Handholding Is Associated With Pain Reduction.” Proceedings of the National Academy of Sciences 115, no. 11: 2528–2537. 10.1073/pnas.1703643115. PMC5856497 29483250

[brb371683-bib-0025] Grinsted, A. , J. C. Moore , and S. Jevrejeva . 2004. “Application of the Cross Wavelet Transform and Wavelet Coherence to Geophysical Time Series.” Nonlinear Processes in Geophysics 11, no. 5/6: 561–566. 10.5194/npg-11-561-2004.

[brb371683-bib-0026] Hasson, U. , A. A. Ghazanfar , B. Galantucci , S. Garrod , and C. Keysers . 2012. “Brain‐to‐Brain Coupling: A Mechanism for Creating and Sharing a Social World.” Trends in Cognitive Sciences 16, no. 2: 114–121. 10.1016/j.tics.2011.12.007. 22221820 PMC3269540

[brb371683-bib-0027] Hasson, U. , Y. Nir , I. Levy , G. Fuhrmann , and R. Malach . 2004. “Intersubject Synchronization of Cortical Activity During Natural Vision.” Science 303, no. 5664: 1634–1640. 10.1126/science.1089506. 15016991

[brb371683-bib-0028] Heiser, M. , M. Iacoboni , F. Maeda , J. Marcus , and J. Mazziotta . 2003. “The Essential Role of Broca's Area in Imitation.” European Journal of Neuroscience 17: 1123–1128. 10.1046/j.1460-9568.2003.02530.x. 12653990

[brb371683-bib-0029] Higuchi, S. , H. Holle , N. Roberts , S. B. Eickhoff , and S. Vogt . 2012. “Imitation and Observational Learning of Hand Actions: Prefrontal Involvement and Connectivity.” Neuroimage 59, no. 2: 1668–1683. 10.1016/j.neuroimage.2011.09.021. 21983182

[brb371683-bib-0067] Holper, L. , F. Scholkmann , and M. Wolf . 2012. “Between‐brain Connectivity During Imitation Measured by fNIRS.” Neuroimage 63, no. 1: 212–222. 10.1016/j.neuroimage.2012.06.028. 22732563

[brb371683-bib-0030] Hu, Y. , Y. Hu , X. Li , Y. Pan , and X. Cheng . 2017. “Brain‐to‐Brain Synchronization Across Two Persons Predicts Mutual Prosociality.” Social Cognitive and Affective Neuroscience 12, no. 12: 1835–1844. 10.1093/scan/nsx118. 29040766 PMC5716073

[brb371683-bib-0031] Huppert, T. J. , S. G. Diamond , M. A. Franceschini , and D. A. Boas . 2009. “HomER: A Review of Time‐Series Analysis Methods for Near‐Infrared Spectroscopy of the Brain.” Applied Optics 48, no. 10: D280. 10.1364/ao.48.00d280. 19340120 PMC2761652

[brb371683-bib-0032] Iacoboni, M. 2009. “Imitation, Empathy, and Mirror Neurons.” Annual Review of Psychology 60, no. 1: 653–670. 10.1146/annurev.psych.60.110707.163604. 18793090

[brb371683-bib-0033] Iacoboni, M. , R. P. Woods , M. Brass , H. Bekkering , J. C. Mazziotta , and G. Rizzolatti . 1999. “Cortical Mechanisms of Human Imitation.” Science 286, no. 5449: 2526–2528. 10.1126/science.286.5449.2526. 10617472

[brb371683-bib-0034] Jackson, P. L. , A. N. Meltzoff , and J. Decety . 2006. “Neural Circuits Involved in Imitation and Perspective‐Taking.” Neuroimage 31, no. 1: 429–439. 10.1016/j.neuroimage.2005.11.026. 16406257 PMC1475952

[brb371683-bib-0064] Kessler, K. , and L. A. Thomson . 2010. “The Embodied Nature of Spatial Perspective Taking: Embodied Transformation versus Sensorimotor Interference.” Cognition 114, no. 1: 72–88. 10.1016/j.cognition.2009.08.015. 19782971

[brb371683-bib-0035] Kostorz, K. , V. L. Flanagin , and S. Glasauer . 2020. “Synchronization Between Instructor and Observer When Learning a Complex Bimanual Skill.” Neuroimage 216: 116659. 10.1016/j.neuroimage.2020.116659. 32119985

[brb371683-bib-0036] Krüger, B. , M. Bischoff , C. Blecker , et al. 2014. “Parietal and Premotor Cortices: Activation Reflects Imitation Accuracy During Observation, Delayed Imitation and Concurrent Imitation.” Neuroimage 100: 39–50. 10.1016/j.neuroimage.2014.05.074. 24907485

[brb371683-bib-0037] Leng, X. , W. Zhu , R. E. Mayer , and F. Wang . 2024. “The Viewing Perspective Effect in Learning From Instructional Videos: A Replication and Neuroimaging Extension.” Learning and Instruction 94: 102004. 10.1016/j.learninstruc.2024.102004.

[brb371683-bib-0038] Liu, J. , R. Zhang , B. Geng , et al. 2019. “Interplay Between Prior Knowledge and Communication Mode on Teaching Effectiveness: Interpersonal Neural Synchronization as a Neural Marker.” Neuroimage 193: 93–102. 10.1016/j.neuroimage.2019.03.004. 30851445

[brb371683-bib-0039] Mahmoudzadeh, M. , G. Dehaene‐Lambertz , M. Fournier , et al. 2013. “Syllabic Discrimination in Premature Human Infants Prior to Complete Formation of Cortical Layers.” Proceedings of the National Academy of Sciences 110, no. 12: 4846–4851. 10.1073/pnas.1212220110. PMC3607062 23440196

[brb371683-bib-0040] Meltzoff, A. N. 2005. “Imitation and Other Minds: The “Like Me” Hypothesis.” Perspectives on Imitation: From Neuroscience to Social Science 2: 55–77.

[brb371683-bib-0041] Meltzoff, A. N. 2007. “The ‘Like Me’ Framework for Recognizing and Becoming an Intentional Agent.” Acta Psychologica 124, no. 1: 26–43. 10.1016/j.actpsy.2006.09.005. 17081488 PMC1852490

[brb371683-bib-0042] Meltzoff, A. N. 1988. “Infant Imitation and Memory: Nine‐Month‐Olds in Immediate and Deferred Tests.” Child Development 59, no. 1: 217. 10.2307/1130404. 3342714 PMC3652622

[brb371683-bib-0043] Meltzoff, A. N. , and R. A. Williamson . 2013. “Imitation: Social, Cognitive, and Theoretical Perspectives.” In The Oxford Handbook of Developmental Psychology, Vol. 1: Body and Mind, edited by P. D. Zelazo , 651–682. Oxford University Press. 10.1093/oxfordhb/9780199958450.013.0023.

[brb371683-bib-0044] Miller, E. K. , and J. D. Cohen . 2001. “An Integrative Theory of Prefrontal Cortex Function.” Annual Review of Neuroscience 24: 167–202. 10.1146/annurev.neuro.24.1.167. 11283309

[brb371683-bib-0045] Nozawa, T. , Y. Sasaki , K. Sakaki , R. Yokoyama , and R. Kawashima . 2016. “Interpersonal Frontopolar Neural Synchronization in Group Communication: An Exploration Toward fNIRS Hyperscanning of Natural Interactions.” Neuroimage 133: 484–497. 10.1016/j.neuroimage.2016.03.059. 27039144

[brb371683-bib-0046] Pan, Y. , S. Dikker , P. Goldstein , Y. Zhu , C. Yang , and Y. Hu . 2020. “Instructor‐Learner Brain Coupling Discriminates Between Instructional Approaches and Predicts Learning.” Neuroimage 211: 116657. 10.1016/j.neuroimage.2020.116657. 32068165

[brb371683-bib-0069] Pan, Y. , X. Cheng , and Y. Hu . 2023. “Three Heads Are Better Than One: Cooperative Learning Brains Wire Together When a Consensus Is Reached.” Cerebral Cortex 33, no. 4: 1155–1169. 10.1093/cercor/bhac127. 35348653

[brb371683-bib-0047] Rizzolatti, G. , and L. Craighero . 2004. “The Mirror‐Neuron System.” Annual Review of Neuroscience 27, no. 1: 169–192. 10.1146/annurev.neuro.27.070203.144230. 15217330

[brb371683-bib-0065] Samuel, S. , T. M. Erle , L. P. Kirsch , et al. 2024. “Three Key Questions to Move towards a Theoretical Framework of Visuospatial Perspective Taking.” Cognition 247: 105787. 10.1016/j.cognition.2024.105787. 38583320

[brb371683-bib-0068] Shadaydeh, M. , V. Noering , M. Franz , T. Chand , I. Croy , and J. Denzler . 2025. “Directionality of Interpersonal Neural Influence in Functional Near‐Infrared Spectroscopy Hyperscanning: Feasibility of Information–Theoretic Causality Analysis in Motor Tasks.” European Journal of Neuroscience 62, no. 6: e70252. 10.1111/ejn.70252. 40994030 PMC12461139

[brb371683-bib-0048] Shea, C. H. , D. L. Wright , G. Wulf , and C. Whitacre . 2000. “Physical and Observational Practice Afford Unique Learning Opportunities.” Journal of Motor Behavior 32, no. 1: 27–36. 10.1080/00222890009601357. 11008269

[brb371683-bib-0049] Singh, A. K. , M. Okamoto , H. Dan , V. Jurcak , and I. Dan . 2005. “Spatial Registration of Multichannel Multi‐Subject fNIRS Data to MNI Space Without MRI.” Neuroimage 27, no. 4: 842–851. 10.1016/j.neuroimage.2005.05.019. 15979346

[brb371683-bib-0050] Skinner, A. L. , K. R. Olson , and A. N. Meltzoff . 2020. “Acquiring Group Bias: Observing Other People's Nonverbal Signals Can Create Social Group Biases.” Journal of Personality and Social Psychology 119, no. 4: 824–838. 10.1037/pspi0000218. 31524429 PMC8276637

[brb371683-bib-0051] Sowden, S. , and C. Catmur . 2015. “The Role of the Right Temporoparietal Junction in the Control of Imitation.” Cerebral Cortex 25, no. 4: 1107–1113. 10.1093/cercor/bht306. 24177989 PMC4380005

[brb371683-bib-0052] Su, W. C. , M. Culotta , J. Mueller , D. Tsuzuki , K. Pelphrey , and A. Bhat . 2020. “Differences in Cortical Activation Patterns During Action Observation, Action Execution, and Interpersonal Synchrony Between Children With or Without Autism Spectrum Disorder (ASD): An fNIRS Pilot Study.” PLoS ONE 15, no. 10: e0240301. 10.1371/journal.pone.0240301. 33119704 PMC7595285

[brb371683-bib-0053] Sun, M. X. B. 2022. Abacus Skills. Higher Education Press.

[brb371683-bib-0054] Sweller, N. , A. Shinooka‐Phelan , and E. Austin . 2020. “The Effects of Observing and Producing Gestures on Japanese Word Learning.” Acta Psychologica 207: 103079. 10.1016/j.actpsy.2020.103079. 32422417

[brb371683-bib-0055] Torrence, C. , and G. P. Compo . 1998. “A Practical Guide to Wavelet Analysis.” Bulletin of the American Meteorological Society 79, no. 1: 61–78. 10.1175/1520-0477(1998)079<0061:APGTWA>2.0.CO;2.

[brb371683-bib-0056] Tsuzuki, D. , V. Jurcak , A. K. Singh , M. Okamoto , E. Watanabe , and I. Dan . 2007. “Virtual Spatial Registration of Stand‐Alone fNIRS Data to MNI Space.” Neuroimage 34, no. 4: 1506–1518. 10.1016/j.neuroimage.2006.10.043. 17207638

[brb371683-bib-0057] Vogt, S. , G. Buccino , A. M. Wohlschläger , et al. 2007. “Prefrontal Involvement in Imitation Learning of Hand Actions: Effects of Practice and Expertise.” Neuroimage 37, no. 4: 1371–1383. 10.1016/j.neuroimage.2007.07.005. 17698372

[brb371683-bib-0058] Watanabe, R. , T. Higuchi , Y. Kikuchi , and M. Taira . 2017. “Visuomotor Effects of Body Part Movements Presented in the First‐Person Perspective on Imitative Behavior.” Human Brain Mapping 38, no. 12: 6218–6229. 10.1002/hbm.23823. 28929542 PMC6867061

[brb371683-bib-0059] Weeks, D. L. , A. K. Hall , and L. P. Anderson . 1996. “A Comparison of Imitation Strategies in Observational Learning of Action Patterns.” Journal of Motor Behavior 28, no. 4: 348–358. 10.1080/00222895.1996.10544604. 14769556

[brb371683-bib-0060] Zheng, L. , C. Chen , W. Liu , et al. 2018. “Enhancement of Teaching Outcome Through Neural Prediction of the Students' Knowledge State.” Human Brain Mapping 39, no. 7: 3046–3057. 10.1002/hbm.24059. 29575392 PMC6866636

[brb371683-bib-0061] Zhidan, W. , A. N. Meltzoff , and R. A. Williamson . 2015. “Social Learning Promotes Understanding of the Physical World: Preschool Children's Imitation of Weight Sorting.” Journal of Experimental Child Psychology 136: 82–91. 10.1016/j.jecp.2015.02.010. 25866145

[brb371683-bib-0062] Zhidan, W. , R. A. Williamson , and A. N. Meltzoff . 2015. “Imitation as a Mechanism in Cognitive Development: A Cross‐Cultural Investigation of 4‐Year‐Old Children's Rule Learning.” Frontiers in Psychology 6: 562. 10.3389/fpsyg.2015.00562. 26029132 PMC4429617

[brb371683-bib-0063] Zhu, Y. , V. Leong , Y. Hou , D. Zhang , Y. Pan , and Y. Hu . 2022. “Instructor–Learner Neural Synchronization During Elaborated Feedback Predicts Learning Transfer.” Journal of Educational Psychology 114, no. 6: 1427–1441. 10.1037/edu0000707.

