# Supplementary material for: Brain‐to‐Brain Coupling Is Associated With the Benefits of Delayed Imitation in Skill Acquisition: An fNIRS Study
Source: Brain Behav. 2026 Aug 20;16(8):e71683. doi: 10.1002/brb3.71683 (PMC13493701; doi:10.1002/brb3.71683)
Supplement: Supplementary file 1 [file BRB3-16-e71683-s001.docx]

**Supplementary Material: Control Analyses of Adjacent Frequency Bands**

To confirm that the interpersonal brain synchronization (IBS) observed in the 0.53 to 0.67 Hz band reflects neural processes rather than systemic physiological noise, we performed control analyses on three additional frequency bands. These included two adjacent bands (0.40 to 0.53 Hz and 0.67 to 0.80 Hz) and a typical respiratory band (0.15 to 0.30 Hz). We applied the same permutation test procedure to the regions of interest (ROIs) that showed significant IBS in the previous analysis.

The permutation tests revealed that the control bands did not exhibit the consistent task-related IBS observed in the primary frequency band (Figure S1). Specifically, the permutation tests yielded non-significant results for the 0.15–0.30 Hz band (*p*s > 0.74), the 0.40–0.53 Hz band (all *p*s > 0.08), and the 0.67–0.80 Hz band (all *p*s > 0.67). These results provide evidence that the task-related synchronization patterns observed in our primary analysis are frequency-specific and are highly unlikely to be driven by broad physiological artifacts such as respiration or cardiac pulsations.

**Figure S1. The ROI-based permutation analysis for the control frequency bands.** No significant IBS enhancement was observed for true dyads compared to pseudo-dyads in any of the control bands: (A–D) the 0.15–0.30 Hz band, (E–H) the 0.40–0.53 Hz band, and (I–L) the 0.67–0.80 Hz band across the Right OFC/FPA, Right dlPFC, Left dlPFC, and Left SMC.
